# Impact of Self-Efficacy on Entrepreneurs’ Ambidextrous Behavior in New Ventures: Moderating Effect of Status

**DOI:** 10.3390/bs13020108

**Published:** 2023-01-28

**Authors:** Jun Ma, Yuzhen Duan, Jianan Wang, Mengjie Luo

**Affiliations:** 1 The College of Humanities, Shandong Management University, Jinan 250357, China; 2 College of Economics and Management, Anhui Open University, Hefei 230022, China; 3 Department of History of Science and Scientific Archaeology, University of Science and Technology of China, Hefei 230026, China; 4 Research Center for Science and Technology Innovation and Regional Development, University of Science and Technology of China, Hefei 230026, China; 5 College of Business, City University of Hong Kong, Hong Kong 999077, China

**Keywords:** entrepreneurial self-efficacy, entrepreneurs’ ambidextrous behavior, status, new ventures

## Abstract

This article discusses the mechanism of the ambidextrous behavior of entrepreneurs in exploring and exploiting simultaneously in new ventures. We draw on social cognition theory to discuss the influence of entrepreneurial self-efficacy (ESE) on entrepreneurs’ ambidextrous behavior and the moderating effect of their status. We contend that an inverted ‘U’ relationship exists between ESE and entrepreneurs’ ambidextrous behavior. A higher economic status of an entrepreneur strengthens the relationship between ESE and that entrepreneur’s ambidextrous behavior, whereas higher power status weakens the relationship. Analyses of high-tech industry entrepreneurs support our hypotheses in the context of emerging economies, represented by China.

## 1. Introduction

Ambidexterity, the ability to explore and exploit simultaneously, has been widely discussed in the literature on management [1,2,3,4,5,6]. Entrepreneurs are the authoritative managers of their organizations, so their behavioral capacity, both to explore through the ‘search for new, useful adaptations’ and to exploit through ‘the use and propagation of known adaptations’ [1,7,8], is vital to the survival and performance of the organization.

Much literature and research have focused on organizational ambidexterity and paradoxical tensions between exploration and exploitation from organizational perspectives [1,9]. Many paradigms and constructs are developed to describe them, such as organizational learning [10], strategic management [11,12], organizational design [13,14], and organizational ambidextrous dynamic capabilities [15,16]. In recent years, ambidextrous behavior at the individual level has also received attention from many scholars. We are aware that some studies provide practical examples of the ambidextrous behavior of managers [17,18] or top management team’s heterogeneity [9,19]. Mom et al., (2009) contributed to this effort by “proposing and clarifying three related characteristics of ambidextrous managers: host contradictions, multitaskers, and both refine and renew the knowledge, skills, and expertise” ([20] p. 812). Since the relationship between individual behavior and individual psychology is mutual correspondence and mutual influence, we developed a proportion that combining “exploration and exploitation” with some critical factors from psychological theories can better reveal the entrepreneur’s status in decision-making.

From a social cognitive theory perspective, this paper investigates how entrepreneurial self-efficacy (ESE) influences entrepreneurs’ ambidextrous activities in new ventures. In this research, we introduce the core concept of social cognitive theory—self-efficacy and demonstrate how it plays a vital role in determining entrepreneurs’ choices and efforts and becomes a critical motivational factor for individuals to engage in ambidextrous behavior [21,22]. Due to fierce market competition, entrepreneurs make full use of their scarce resources, so maintaining an effective balance between robust exploitative innovations and breakthrough exploratory innovations is vital to the survival and development of enterprises in the marketplace [1], which also confirmed by Wang’s research regarding open innovation and to survive of large high-tech enterprises [23]. Suppose entrepreneurs with high ESE are determined to implement challenging ambidextrous behaviors. They are willing to engage in new and creative practices [24]. They firmly believe they can seize opportunities under challenging circumstances and balance exploitative and exploratory activities [22], enabling their companies to gain competitive advantages. However, a competency trap can occur when good performance with an inferior procedure leads an organization to accumulate more experience, thus keeping experience with a superior procedure inadequate to recognize its superiority [25]. Such behavior will limit the efforts of enterprises to change their current core capabilities [10] and constrain entrepreneurs’ ambidextrous behavior.

The status of entrepreneurs can help them analyze and judge their position in a complex market environment. As a result, cognitive differences regarding their abilities are generated. This situation may influence individual learning, knowledge gathering, information processing, and decision-making preferences, all of which impact entrepreneurs’ critical behaviors in the future [26]. At the same time, as the leaders of organizational activities, entrepreneurs of new ventures can take advantage of their status in the early stage of enterprise development, attract a large number of resources for the enterprise, and make up for the innate deficiencies of new ventures.

Our study makes three significant contributions. Firstly, it goes beyond previous research, primarily on organizational ambidexterity. We find such studies lack conceptually and empirically validated understanding of entrepreneur ambidexterity because adequately determining the antecedents of entrepreneur ambidexterity in an individual’s psychological level was not or not sufficiently discussed in the existing literature [9,15,16,27]. Therefore, an individual perspective to clarify the ambidextrous mechanisms is developed in our study to contribute to the literature. Specifically, we show that ESE impacts entrepreneurs’ contradictory exploration- and exploitation-related behavior. Secondly, more research needs to disentangle how individual status moderate the main effect. The current work discusses the role of entrepreneurs’ status in the market and the promotion and inhibition of entrepreneurs’ individual cognition and ambidextrous behavior in new ventures. Scholars have studied the relationship between individual status and innovation performance, but only some have discussed the relationship between individual status and ambidextrous behavior [28]. Thirdly, the research was conducted in the context of emerging economies, using China as a representative. Individuals in different regions may have significant cognitive differences due to the influence of history and culture. Political power is vital in developing a market economy, especially in China. The power status of entrepreneurs in China may significantly affect the choice of strategy in a way that differs from that impact in Western countries.

High-technology enterprises as the sampling frame to test our thesis because these high-tech enterprises are considered knowledge- and technology-intensive enterprises. Firstly, Tensions and trade-offs escalate when high-tech enterprises attempt to implement strategic, product, or market ambidexterity, which is executed within a single functional domain. Compared with other companies, high-tech enterprises face a more uncertain external environment [29] and have a stronger incentive to develop new and competitive products for the marketplace [23,30]. Secondly, entrepreneurs of high-tech enterprises generally face competitive pressures to combine exploration and exploitation, doing both concurrently, which brings the entrepreneurs of high-tech enterprises difficulties in managing and coordinating conflicting knowledge processes (structural ambidexterity).

## 2. Theoretical Background and Hypothesis Development

Paradoxical decision processes between exploration and exploitation imply the value of individual ambidexterity [6,10,31,32]. The relevance of investigating an entrepreneur’s ambidexterity is emphasized by studies that discuss an entrepreneur’s ability to become ambidextrous in terms of, for instance, development routine and alliance strategy [7,20,33]. First, entrepreneurs frequently must deal with ambidextrous risks and opportunities [13,31] and engage in paradoxical thinking [34]. Second, entrepreneurs fulfill multiple roles related to both competence deployment and competence definition activities [31], typically act outside the narrow confines of their job [34], and conduct routine activities [33]. Third, entrepreneurs must rapidly refine and renew their knowledge, skills, expertise, and social network ties [35].

### 2.1. Entrepreneur Self-Efficacy and Ambidextrous Behavior

In general, self-efficacy is cultivated in complex cognitive, psychological, and social skills through the individual’s experience accumulation or repeated achievement [36,37]. The accomplishment of a difficult task through perseverance will provide individuals with positive self-efficacy, which will continue to increase with more accumulation of successful experiences. Such self-efficacy will give individuals the confidence to set further challenging goals for themselves [22]. New ventures have limited resources in the early stages of development, and exploitative and exploratory activities will compete for scarce resources. Due to fierce market competition, start-ups make full use of their existing resources, so maintaining an effective balance between robust exploitative innovations and breakthrough exploratory innovations is vital to the survival and development of enterprises [1]. If start-ups try to maximize both types of innovation, then they will face enormous resource challenges. Entrepreneurs with high ESE thrive on such challenges and are willing to engage in new and creative practices [24] to solve problems innovatively [26]. Thus, entrepreneurs with high ESE tend to lead enterprises to implement ambidextrous behaviors.

Entrepreneurs’ sense of self-efficacy can affect their entrepreneurial decision-making and opportunity identification in many ways [24,36]. For example, self-efficacy is critical in determining how entrepreneurs search for and acquire new opportunities. As a result, entrepreneurs with high self-efficacy tend to focus on exploring new businesses with positive beliefs, while those with low self-efficacy tend to focus on what may go wrong. Their successful experience accumulated in the past enables entrepreneurs to achieve improved self-efficacy [24]. This positive belief will make them self-adjust in adversity, recover quickly, and insist on completing the original goal [24,37,38]. Therefore, entrepreneurs with high ESE are determined to implement challenging ambidextrous behaviors. They will maintain their efforts and firmly believe they can seize opportunities under challenging circumstances and balance exploitative and exploratory activities, enabling their companies to gain core competitive advantages. However, entrepreneurs with low ESE may have a minimal successful experience and lack accumulated positive self-efficacy. As a result, they may worry about resource limitations and environmental instability; they fear that they may ineffectively allocate resources. This situation leads to the failure of ambidextrous behavior, which may expose companies to the risk of declining performance and even bankruptcy. To avoid the risks above, entrepreneurs may temporarily break the balance between exploration and exploitation activities and choose strategies of low risk to meet the company’s primary development needs.

However, when the ESE of entrepreneurs reaches a very high level and exceeds a certain threshold, the ambidextrous behavior intention of entrepreneurs with high ESE may be decreased. As we mentioned above, individuals mainly obtain self-efficacy through successful experience accumulation or repeated achievements. When ESE exceeds a certain threshold, entrepreneurs have accumulated a profound experience of success. Leonard-Barton (1992) argued that when the environment changes and companies need to develop new capabilities [39], companies will rely on long-established fixed models and gradually generate core rigidity that hinders corporate change. We think that not only enterprises will have core rigidity in the development process but also entrepreneurs; as the core individuals in enterprises, they will form path dependence through long-term experience accumulation, thereby leading to core rigidity of entrepreneurs. That is, entrepreneurs with several successful experiences rely upon their actual knowledge and experience when faced with changes in the external environment, and they are convinced those will suffice because of past success. However, a competency trap can occur when good performance with an inferior procedure leads an organization to accumulate more experience, thus keeping experience with a superior procedure inadequate to recognize its superiority [25]. Such behavior will limit the efforts of enterprises to change their current core capabilities and constrain entrepreneurs’ exploratory behavior [10]. From the perspective of early experiences and myopic search, people’s myopic tendencies in search behavior are common when facing uncertainty; this phenomenon indicates that past experiences induce people to search only options close to the original plan, and they choose the solution closest to these experiences [40]. Therefore, when entrepreneurs gain sufficient successful experience and ESE reaches a high level, they may be paralyzed by their previous successful experiences and disinclined to seek improved methods. The latter will hinder them from making breakthroughs and changes and weaken their ambidextrous behavior. Thus, we offer the following hypothesis:

**H1.**  *An inverted ‘U’ relationship exists between ESE and entrepreneurs’ ambidextrous behavior. Up to a determinable point, increments in ESE are positively related to entrepreneurs’ ambidextrous behavior. However, further increments in ESE are negatively related to entrepreneurs’ ambidextrous behavior.*

### 2.2. Moderating Effect of Status

Washington and Zajac defined “status” as a ‘socially constructed, intersubjectively agreed-upon and accepted ordering or ranking of individuals, groups, organizations or activities in a social system’ [41]. Expectation state theory proposes that status characteristics are the basis for inferring the individual’s ability and expected performance. Generally, a person with higher status has a more remarkable ability to succeed than someone with lower status [42]. We divide status into the economic status and power status. Wealth reflects people’s unequal status in the economic field, and the amount of power distributed and possessed reflects people’s unequal status in the political field [43].

#### 2.2.1. Economic Status

Funding support is an essential foundation for any firm’s development and innovation. Entrepreneurs with ample funds can successfully implement ambidextrous behavior. As the principal founders and the leaders in production and business activities in new ventures, entrepreneurs with higher self-efficacy and higher economic status will be more inclined to implement ambidextrous behaviors.

Before the ESE of entrepreneurs reaches a very high level and exceeds a certain threshold, if entrepreneurs have high economic status and high ESE, they have great strength to implement ambidextrous behavior, which will further strengthen the motivation for their ambidextrous behavior. This situation is attributed to two factors. Firstly, entrepreneurs with high economic status have high family incomes and household assets. Their families may provide enterprises with substantial financial support, such as raising the registered capital of enterprises, purchasing the technology, hiring professional talents, and safeguarding multichannel corporate financing. These supports will enhance the supply of innovative elements such as corporate capital, technology, and talent. This increase in innovation power will undoubtedly encourage the ambidextrous motivation of entrepreneurs with high ESE. Secondly, given that family income is positively correlated with the size and network diversity of the embedded network [44], the extensive network size and network diversity indicate the richness and heterogeneity of network resources [45]. Entrepreneurs with high economic status can obtain rich and heterogeneous network resources. These network resources not only can compensate for the inherent deficiencies in the scarcity of innovation resources of start-ups but also enable entrepreneurs to use their rich network resources to attract other market players for cooperation or alliances. Cooperating with partners, especially famous companies, can convey signals about company resources and prospects [46]. Therefore, cooperation or alliances with other companies will bring further resources to new ventures. Such resource advantages brought by high economic status will further stimulate entrepreneurs with high self-efficacy, so before the ESE of entrepreneurs reaches a very high level and exceeds a certain threshold, a higher economic status is a positive catalyst for entrepreneurs to exert self-efficacy and achieve ambidextrous innovation.

However, when ESE reaches a high level and exceeds a certain threshold, entrepreneurs have accumulated a wealth of successful experience that can make the core rigid. Entrepreneurs will subconsciously apply and strengthen the original, successful model when they face changes in the external environment. Excessive reliance on experience will hinder entrepreneurs’ exploratory behavior. At this time, the assets and network resources owned by the company make the entrepreneurs highly convinced that past behavior will be best because most of these assets and resources were obtained by that behavior. Overconfidence and excessive use of past techniques will hinder entrepreneurs’ exploratory behavior. The resources brought by the high economic status will have overlapping effects with the original resource accumulation experience. This effect is not enough to generate incentives for entrepreneurs with high self-efficacy. However, it will strengthen the negative correlation between entrepreneur self-efficacy and ambidextrous innovation behavior because entrepreneurs worry about the risk-taking nature of ambidextrous innovations and think it could endanger their high economic status. Thus, we provide the following hypothesis:

**H2.**  *The economic status of entrepreneurs moderates the relationship between ESE and entrepreneurs’ ambidextrous behavior. A high economic status strengthens the inverted ‘U’ relationship between ESE and entrepreneurs’ ambidextrous behavior.*

#### 2.2.2. Power Status

Weber (1947) regarded power as the primary basis for stratification in the political field when he described the social hierarchy [43]; the differing power status of individuals will reflect their inequality in this aspect. Power status further reflects the ‘social importance’ of the individual, especially in Chinese culture, which is characterized by high power distance. In such a culture, a high-power status weakens the relationship between ESE and entrepreneurs’ ambidextrous behavior. The market and government allocation mechanisms exist simultaneously in emerging economies such as China [47,48]. The government largely dominates the allocation of resources and information [49,50], such as bank loans, land, and tax incentives [51]. Both the Civil Servants Act of China and the Regulations of the CPC on Disciplinary Punishment regulate those government officers should not engage in profit-making activities and work part-time for enterprises or other profit-making organizations. In this situation, we addressed the preliminary separation between the two research objects, government officers and entrepreneurs, and kept the identity independence among each other. Even as mentioned above, many entrepreneurs can still gain access to policy information and acquire legitimacy from the government to benefit their businesses. Many entrepreneurs who are deputies to the National People’s Congress (NPC) and members of the Chinese People’s Political Consultative Conference (CPPCC) of each city, province, or state have high power status and are politically connected with the government and can obtain political resources. Under the influence of China’s ‘official standard’ thinking, entrepreneurs with high ESE may focus on maintaining and acquiring their personal power status and political resources. They seek to improve the legitimacy of enterprises and obtain opportunities for promotion, which may compete with their motivation for ambidextrous behavior. The reason is that, although entrepreneurs focus on acquiring political resources, they face the risk of ‘embedding’. Highly embedding the entrepreneur in the political network may consume a significant amount of time and money and have other hidden costs [52], resulting in the loss of innovative resources. Therefore, entrepreneurs with high ESE will be inclined to invest in the scarce resources of enterprises to obtain a large number of political connections, and the resulting lack of resources reduces the motivation for ambidextrous behavior.

Existing research also shows that political power resources can facilitate the legitimacy of enterprises, commercial access, financing constraints, tax evasion, and even increase enterprise value [50,51], which may be helpful to innovation. When ESE reaches a high level and exceeds a certain threshold, entrepreneurs have accumulated rich innovation resources, increased market initiative, and weakened the restraint of government coercion. At this point, the political resources brought by the power position will encourage entrepreneurs with a high sense of self-efficacy and thus weaken the negative correlation between entrepreneur self-efficacy and ambidextrous innovation behavior. Thus, we offer the following hypothesis:

**H3.**  *The power status of entrepreneurs moderates the relationship between ESE and entrepreneurs’ ambidextrous behavior. A high power status will weaken the inverted ‘U’ relationship between ESE and entrepreneurs’ ambidextrous behavior.*

**Figure 1:**
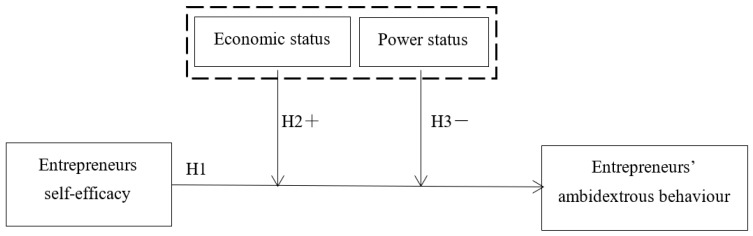
Theoretical model.

Figure 1 shows the theoretical model of this article.

## 3. Method

### 3.1. Sample

This study’s questionnaire and objective cross-section data are drawn from entrepreneurs running high-technology enterprises in China. These high-tech enterprises all are considered knowledge- and technology-intensive enterprises. Compared with other companies, high-tech enterprises face higher environmental uncertainty and have a stronger incentive to develop new products [30,52]. McDougall & Robinson (1990) defined “new ventures” as enterprises that have been established for less than eight years [53]. Forbes (2005) regarded entrepreneurs as individuals who have created their businesses [54], whereas Gartner (1985) viewed entrepreneurs as founders of new organizations [55]. Consistent with previous research [56,57,58,59], we define an entrepreneur as an individual who (a) is responsible for the independent or collaborative starting up a new business, and (b) is currently the prominent owner and manager of the new venture. Entrepreneurs are in a dominant position to affect innovation.

To adapt to the Chinese context, we used the Back Translation method proposed by Brislin (1980) to compose the questionnaire in Chinese to establish a genuine delivery of the measurement content [60]. We took measures to obtain objective and accurate results when collecting data. The data with certain control variables (e.g., industry, firm size, entrepreneur gender, and age) and moderator variables (economic status and power status of entrepreneurs) were collected from cross-section data obtained from the industrial and commercial bureaus of different regions. The questionnaire consisted of two main parts: basic information and investigation. In the investigation part, we referred to McGee’s design in Entrepreneur’s Self-efficacy and Lubatkin’s design in ambidextrous behavior with some necessary modifications [61,62]. The full text of the questionnaire in the English version was attached in Appendix A.

Our study randomly selected entrepreneurs from new ventures in high-tech enterprises to participate in the study. To check the survey’s validity, a pilot test was conducted with 55 entrepreneurs participating in an advanced management-training program (2019) in Hefei, China. Some modifications to the wording were made based on their feedback. The sample interviews and the pilot study were obtained using the snowball approach. Then, we distributed questionnaires to entrepreneurs in a random sample, focusing on Jiangsu, Zhejiang, Shanghai, Anhui, and Beijing provinces or municipalities of China. The questionnaire data from Anhui Province were collected from MBA graduates at the University of Science and Technology of China (in Anhui). Additionally, we also entrusted our friends at Beihang University (in Beijing), Suzhou University (in Jiangsu), Tongji University (in Shanghai), and Zhejiang Gongshang University (in Zhejiang) to spread the same questionnaires to MBA graduates of each university in order to collect data from these provinces. We collected the data using both paper and electronic questionnaires (through wjx.com, an online questionnaire distributor).

After two rounds of follow-up reminders and the distribution of 350 questionnaires, 227 available questionnaires (n = 227) were received, representing a 64.86% available response rate. The sample included advanced manufacturing (46.26%), high-tech service (25.99%), and other high-tech industries (27.75%). A total of 93.39% of the participating entrepreneurs were male, and the largest group (34.36%) was aged 35 to 45.

### 3.2. Measures

#### 3.2.1. Dependent Variables

**Ambidextrous behavior.** Mom (2009) measured managers’ ambidexterity in two dimensions: exploratory and exploitative activities [20]. This study assumes that entrepreneurs are the leading managers of new ventures. Prior studies have combined exploration and exploitation measures to assess ambidexterity [4,34,62]. Gibson and Birkinshaw (2004) measured ambidexterity by multiplying exploitation and exploration, whereas He and Wong (2004) subtracted exploitation from exploration. Lubatkin et al. (2006) combined exploitation and exploration and thus obtained a lower loss of information than the two other methods. Our study agrees with that of Lubatkin et al. (2006). We use a 14-item Likert scale to measure entrepreneurs’ ambidextrous behavior. All items are measured on a 5-point Likert scale (1 = totally disagree, 2 = disagree, 3 = neutral, 4 = agree, 5 = totally agree). Moreover, a high final score indicates strong entrepreneurs’ ambidextrous behavior. For more details, please see Appendix A.

#### 3.2.2. Independent Variables

**Entrepreneurial self-efficacy (ESE).** We follow McGee et al. (2009) and use the 5-dimension 22-item scale to measure ESE. The scale includes six dimensions: developing new products or markets, creating an innovative atmosphere, developing relationships with investors, defining core goals, coping with unpredictable challenges, and developing core human resources. This part of the survey also uses a 5-point Likert scale (1 = totally disagree, 2 = disagree, 3 = neutral, 4 = agree, 5 = totally agree). A high score by participating entrepreneurs indicates high ESE. For more details, please see Appendix A.

#### 3.2.3. Moderator Variables

**Economic status.** Better economic status usually brings larger choice space and higher risk tolerance to entrepreneurs, so economic status is one important factor affecting business opportunity identification in the decision-making process. Initially, we considered measuring the economic status of individuals by personal income, household income, and household assets. For avoiding research ethics violation, we measured the economic status of entrepreneurs through an indirect approach, which based on the amount of registered capital and the turnover of the enterprise in the past year. In China, most start-ups are founded by entrepreneurs, and a large proportion of these start-ups are family-owned. The companies’ assets are also closely related to the entrepreneur’s assets, and the two will rarely differ significantly. Entrepreneurs with numerous household assets will probably invest a large amount of registered capital in the company. This implies that when the company has a significant turnover, the entrepreneurs will have a better capital situation. Therefore, the registered capital and turnover of the enterprise can be used as a measure of the economic status of entrepreneurs.

We also consider that when many market players decide whether to cooperate or form alliances with new ventures, they will focus on the entrepreneurs’ economic strength to examine the enterprises’ development potential. Given that the entrepreneurs’ family assets involve individual privacy, market players do not have easy access to such information. By contrast, they can quickly obtain reliable data about entrepreneurs’ registered capital and business turnover when they set up enterprises. This information can be used to judge the economic situation of entrepreneurs and consider whether to cooperate or form alliances with new ventures to provide development resources. Considering that the research object of this article is mainly the founders of Chinese high-tech start-ups, we assume the entrepreneur is currently the leading owner and manager of the company.

Moreover, all the data listed above can be legally acquired from national enterprise credit query platforms such as Qichacha (qcc.com, accessed in 31 May 2019) and Tianyancha (tianyancha.com, accessed in 31 May 2019). Thus, the entrepreneur’s economic status can be measured using their registered capital when creating the business and the company’s turnover in the past year. We calculate the economic status of entrepreneurs as the average of the two:

Economic status of entrepreneurs = (enterprise registered capital + business turnover in the past year)/2.

**Power status.** We must carefully consider the connection between politics, business, and the market. Given the reassurance provided by political power, entrepreneurs’ specific political resources will significantly influence whether the enterprise engages in ambidextrous behavior. Among the agents of government in China, individuals who serve as deputies to the National People’s Congress (NPC) or members of the Chinese People’s Political Consultative Conference (CPPCC) are considered to have high political status.

To establish an identification strategy for power status for this research, we refer to Li et al. (2009) and other Chinese scholars’ research on political connections, and measure entrepreneurs’ power status as follows. We measure the power status of entrepreneurs based on their service as NPC deputies or CPPCC members, specifically the government level and the time they have served. We divide the level of NPC deputies or CPPCC members into three levels: municipal level or below (1 point), provincial level (2 points), and national level (3 points). The serving term of NPC deputies or CPPCC members is also divided into three levels: one, two, and three sessions. Similarly, we score 1, 2, and 3 points for each level. We use the average of the two items to indicate the power status of entrepreneurs:

Power status of entrepreneurs = (the scores for the level at which entrepreneurs act as NPC deputies or CPPCC members + the scores for the length of time that entrepreneurs have acted as NPC deputies or CPPCC members)/2.

#### 3.2.4. Control Variables

Prior research has indicated that specific characteristics of individuals and enterprises impact the innovation behavior of entrepreneurs. Our study discusses the entrepreneurs’ ambidextrous behavior at the individual level; thus, the control variables are also selected in individual dimensions to ensure the reliability of our empirical research results. Firstly, we control for the gender and age of entrepreneurs and assume that both factors will impact ambidextrous behavior. In our survey, we use code 1 for males and 0 for females, and we base the age of entrepreneurs on their actual age. Secondly, many studies have indicated that the start-up experience of entrepreneurs can help them make sophisticated judgments that will aid them in predicting performance and setting realistic goals [63,64,65]. We follow Baron (2016).and measure start-up experience using the number of ventures entrepreneurs had previously founded or co-founded [21]. Lastly, the number of years that entrepreneurs serve as enterprise senior managers also significantly influence their ambidextrous behavior. When the management experience of entrepreneurs is high, they have a solid ability to interpret and handle numerous uncertain issues [66]. 

Gibson & Birkinshaw (2004) argued that the innovative skills of ambidextrous managers are mainly based on the accumulation of knowledge and experience in all aspects rather than on their increased professional knowledge [34]. Therefore, the rich management experience of entrepreneurs who are essential managers in enterprises will undoubtedly impact their ambidextrous behavior. To control the management experience of entrepreneurs, we use managerial tenure [18]. 

We also select a few enterprise characteristics as control variables to further ensure the validity of the research results. The survey focuses on high-tech enterprises, which we divide into advanced manufacturing, high-tech service, and other high-tech industries. These we measure with dummy variables. The age and size of the firm will also affect entrepreneurs’ ambidextrous behavior. According to the criteria mentioned above, the surveyed enterprises were all start-ups, having been established for less than eight years. Therefore, we no longer use the age of firms as a control variable but measure firm size by using the number of full-time employees. Our study utilizes the actual number of employees in firms.

## 4. Analysis and Results

Table 1 shows the descriptive statistics and Pearson’s correlation of all variables examined in this study. The correlations of most of the variables in our study are low in magnitude (i.e., below 0.40). The correlation between ESE and entrepreneurs’ ambidextrous behavior is 0.564, but this value remains within the moderate range (i.e., below 0.60); the impact of this result on our analysis is relatively minimal [21]. We also designed several tests for troubleshooting multicollinearity. The typical method for judging multicollinearity is to compare the variance inflation factors (VIFs) to a threshold of 10. We calculate the VIF value for each regression equation, and the highest VIF value is 3.063, which is considerably below the recommended level of 10. As analytical results above, we solved the multicollinearity problem that may affect the robustness of the results.

Moreover, Cronbach’s α values are higher than 0.7, indicating that each variable’s scales have good internal consistency and meet the requirements for further processing. The KMO value is 0.663; the items of the Bartlett Sphericity test loaded significantly (*p* < 0.05), as shown in Table 2. We use confirmatory factor analysis to examine the validity of the scale. The fit indices show that the measurement model fits the data reasonably well (χ^2^ = 611.743; Df = 367; CFI = 0.950; IFI = 0.953; RMSEA = 0.054; RMR = 0.075), which indicates that the model is acceptable. Also, common method deviations can affect many social science studies and cause significant disputes [67,68], so we used Harman’s single -factor test to check for common method bias [69]. The variance contribution rate of the first unrotated factor is 20.101%. This result indicates that the issue of common method bias is acceptable.

Table 3 shows the results of the regression analyses. Model 1 analyses all the control variables. The number of employees (β = 0.106, *p* < 0.10), the gender of entrepreneurs (β = 0.166, *p* < 0.01), and the number of ventures that entrepreneurs had previously founded or co-founded (β = 0.273, *p* < 0.001) are positively related to entrepreneurs’ ambidextrous behavior. In contrast, the age of entrepreneurs is negatively related to entrepreneurs’ ambidextrous behavior (β = −0.177, *p* < 0.01). We add our independent variables in Models 2 and 3. As predicted, the square term of ESE is negative and significant (β = 0.503, *p* < 0.001). This result supports H1 that ESE has an inverted ‘U’ relationship with entrepreneurs’ ambidextrous behavior.

Models 4-1, 5-1, and 6-1 examine the moderating effects of economic status. In Model 4-1, we add economic status. Model 5-1 includes the interaction between economic status and ESE. Model 6-1 includes the interaction between economic status and the square term of ESE. The Model 6-1 results show that the economic status of entrepreneurs moderates the inverted ‘U’ relationship between ESE and entrepreneurs’ ambidextrous behavior (β = 0.143, *p* < 0.05). Therefore, H2 is supported.

Models 4-2, 5-2, and 6-2 examine the moderating effects of power status. We add power status in Model 4-2. Model 5-2 includes the interaction between power status and ESE. Model 6-2 includes the interaction between power status and the square term of ESE. Model 6-2 shows that the power status of entrepreneurs moderates the curvilinear relationship between ESE and entrepreneurs’ ambidextrous behavior (β = −0.232, *p* < 0.01). Thus, H3 is supported.

Using the step-by-step test method [70], this study constructs the following model:

$${\mathrm{EAB}}_{ES,test}={\;\alpha }_{1}\times \mathrm{ESE}\;+{\;\alpha }_{2}\times {\mathrm{ESE}}^{2}+{\;\alpha }_{3}\times \mathrm{ES}\;+{\;\alpha }_{4}\times \mathrm{ES}\times \mathrm{ESE}\;+{\;\alpha }_{5}\times \mathrm{ES}\times {\mathrm{ESE}}^{2}$$

$${\mathrm{EAB}}_{PS,\;test}={\;\beta }_{1}\times \mathrm{ESE}\;+{\;\beta }_{2}\times {\mathrm{ESE}}^{2}+{\;\beta }_{3}\times \mathrm{ES}\;+{\;\beta }_{4}\times \mathrm{ES}\times \mathrm{ESE}\;+{\;\beta }_{5}\times \mathrm{ES}\times {\mathrm{ESE}}^{2}$$

In the above two formulas, *EAB* = entrepreneurs’ ambidextrous behavior; *ESE* = entrepreneurial self-efficacy (‘self-efficacy’ in Table 3); *ES* = economic status; *PS* = power status; and α_n_, β_n_ are the coefficient of variables of Model 6-1 and Model 6-2 in Table 3.

Since the results of regression were standardized, we referred to the previous literature and used the ‘nlcom’ command in Stata 16, showing that the inverted U-shape turns when ES = 1.00 and that the 95% confidence interval for the turning point [0.88, 1.13] is within the value range of ES [71,72]. Similarly, the inverted U-shape turns when PS = 0.56 and that the 95% confidence interval for the turning point [0.55, 0.57] is within the value range of PS.

**Figure 2:**
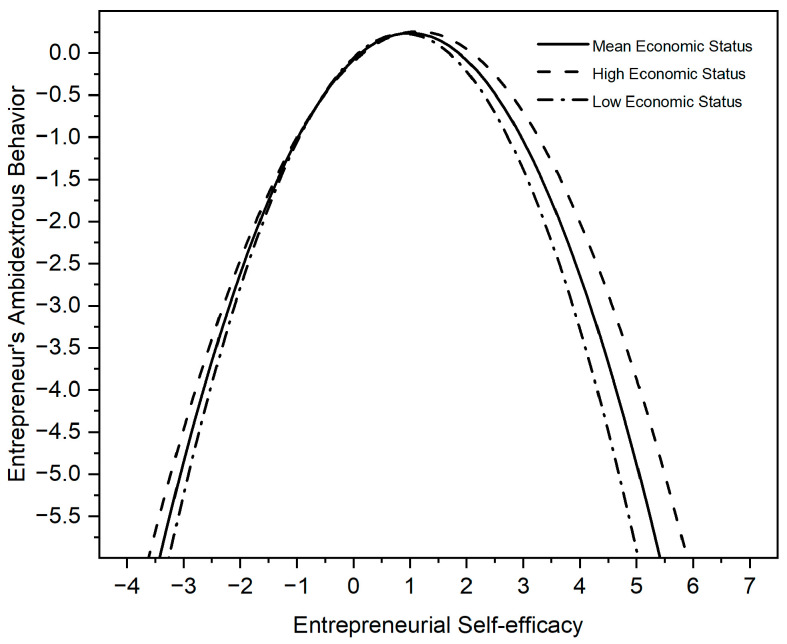
Moderating effects of economic status on self-efficacy and entrepreneurs’ ambidextrous behaviour.

**Figure 3:**
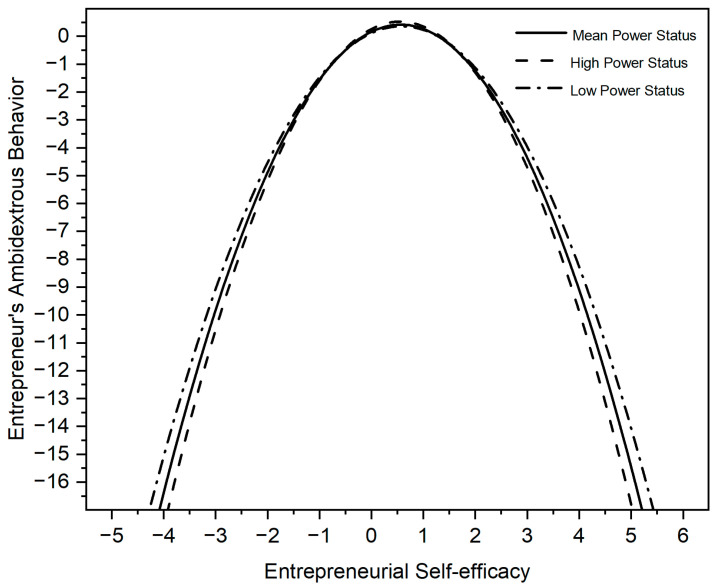
Moderating effects of power status on self-efficacy and entrepreneurs’ ambidextrous behaviour.

The following figures graphically illustrate the inverted ‘U’ relationship between ESE and entrepreneurs’ ambidextrous behavior. Figure 2 illustrates how a higher economic status of entrepreneurs strengthens the effect of ESE on those entrepreneurs’ ambidextrous behavior. Figure 3 illustrates how a higher power status of entrepreneurs weakens the effect of ESE on their ambidextrous behavior.

## 5. Post-Hoc Analyses

We further substantiate our empirical results by conducting additional tests with an alternative measure of the entrepreneurs’ economic status. The results remain qualitatively the same when adopting the entrepreneurs’ salary instead of the enterprise registered capital and business turnover in the past year. The moderating effect of entrepreneurs’ economic status on the curvilinear relationship between ESE and entrepreneurs’ ambidextrous behavior (β = 0.177, *p* < 0.05) remains similar to the previous estimate (Table 4).

## 6. Discussion and Conclusions

How does entrepreneurs’ self-efficacy affect ambidextrous behavior? Although many previous studies have examined the effects of self-efficacy on individual behavior, most scholars believe that a positive relationship exists between them [1,2,3,4,5,6,73]. In our research, we discuss the impact of ESE on entrepreneurs’ ambidextrous behavior in new ventures and propose different views from previous studies. Our results show a significant nonlinear relationship between ESE and entrepreneurs’ ambidextrous behavior in new ventures. Accumulating experience and repeated achievements enable entrepreneurs to cultivate increased self-efficacy, which improves their confidence to set challenging goals and try ambidextrous activities. It also encourages them to adjust their status in adversity until they achieve their goals quickly. However, excessive experience accumulation may cause entrepreneurs to generate core rigidity and fall into the competency trap of relying excessively on experience, which hinders their ambidextrous activities.

For both nascent and experienced entrepreneurs, our study explains why and how they should focus on experience accumulation in practice activities, gradually cultivate self-efficacy, and implement ambidextrous behavior by improving self-awareness to lay the foundation for the future development of the enterprise. To be specific, entrepreneurs should avoid short-sighted search and path dependence while accumulating experience and fostering self-efficacy. They should be brave in implementing exploratory activities to lead corporate change. Accordingly, our research not only enriches the study of the impact of self-efficacy on individual behavior, but also has practical significance for developing entrepreneurs’ ambidextrous behavior in new ventures.

In the past, research on ambidexterity has focused on the organizational level. In recent years, scholars have gradually paid close attention to the study of ambidexterity at the individual level [7,8,74,75]. Previous studies show that successful firms are characterized by ambidexterity [76]. They can maintain the tension between change and evolution and find the balance between them, creating advantages and maintaining advantages, changing and retaining practices, and exploring and exploiting [14]. Start-up high-tech firms are typically small or have been established for only a short time, while founders can significantly impact the firm’s development spontaneously [77,78]. Moreover, the personal characteristics of an entrepreneur are often related to the top management team’s behaviors, especially their active responses to rapidly changing external environments, and are reflected in firm strategy and performance [19,27]. As stated above, there is usually no obstacle for entrepreneurs to implement ambidextrous decisions in new ventures.

From psychological perspectives, Bandura & Locke (2003) pointed out that human behavior is influenced by the expectation of self-behavioral ability and behavior formed by human cognition [79]. Before deciding to carry out an activity and clarifying what action may eventually lead to an excellent result, people often evaluate the whole process of completing the task first [80]. Such behavior is a manifestation of self-efficacy. Thus, we study the influence of ESE on entrepreneurs’ ambidextrous behavior, through which we can analyze the possibility that new ventures implement an ambidextrous innovation strategy. Accordingly, based on social cognitive theory, we discuss the influence of ESE on entrepreneurs’ ambidextrous behavior in new ventures at the individual level and contribute to the research on ambidexterity from the individual perspective. 

The status of entrepreneurs can help them analyze and judge their position in a complex market environment. As a result, it generated cognitive differences in their abilities. However, research is lacking on the influence of individual status on individual ambidextrous behavior. Entrepreneurs with high economic status generally have high family incomes and numerous household assets. Before the ESE of entrepreneurs reaches a very high level and exceeds a certain threshold, such economic factors can provide additional financial support for entrepreneurs’ ambidextrous behavior; they can expand financing channels and attract multiple and heterogeneous innovation resources from the outside. The empirical research results show that the economic status of entrepreneurs and ESE have a solid complementary effect. Entrepreneurs are willing to implement challenging ambidextrous behavior under the influence of ESE. At the same time, high economic support will give entrepreneurs extra strength and resources to consider exploitation and exploration activities, further stimulating entrepreneurs to implement ambidextrous behavior.

When ESE reaches a high level and exceeds a certain threshold, the resources brought by the high economic status will have overlapping effects related to accumulating the original resources. The combined result of the overlapping, opposite effects does not generate enough incentive for ambidexterity for entrepreneurs with high self-efficacy. Entrepreneurs worry about the risk-taking nature of ambidextrous innovations and the danger to their high economic status. Therefore, the high economic status of entrepreneurs strengthens the inverted ‘U’ relationship between ESE and entrepreneurs’ ambidextrous behavior. This result also supplements the research on status.

Meanwhile, prior studies regarding entrepreneurship show that emerging economies in China are quite different from those of Western countries [19,32]. For example, in China, politics and administrative power can interfere with the market economy in many aspects, such as policy preference, tax relief, special subsidies, and license authorization, which usually determine the survival or bankrupt of nascent entrepreneurs and their newborn ventures. Therefore, the perception of power amongst individuals in China may differ from that in the West. The empirical result of this study shows that a high power status will weaken the inverted ‘U’ relationship between ESE and entrepreneurs’ ambidextrous behavior. Entrepreneurs with high power status often have strong political connections to help them in their business. The existence of official standard thought causes entrepreneurs to assign scarce resources from the organization to maintaining political connections, which is, in effect, relinquishing decision-making autonomy. Such actions will meet government requirements and mitigate the risk for entrepreneurs through helping them obtain business legitimacy, policy information, and advancement opportunities easily. Entrepreneurs pursuing the stable development of their enterprises using this approach may weaken their ambidextrous behavior. In other words, the rising power status of entrepreneurs may not promote their ambidexterity.

Finally, our study reveals the significant practical implications of how entrepreneurs of new start-ups in China use political resources to choose different innovation models. When ESE reaches a high level and exceeds a certain threshold, entrepreneurs have accumulated rich innovation resources, increased market initiative, and weakened the restraint of government coercion. At that point, the political resources brought by the power position will encourage entrepreneurs with a high sense of self-efficacy and thus weaken the negative correlation between entrepreneur self-efficacy and ambidextrous innovation behavior.

## 7. Limitations and Future Research

Our study has limitations that provide several directions for future research. Firstly, based on social cognition theory, our study discusses the influence of ESE on individual entrepreneurs’ ambidexterity and concludes that a significant inverted ‘U’ relationship exists. However, we do not specifically discuss the critical value of the relationship between ESE and entrepreneurs’ ambidextrous behavior when individual cognitive rigidity occurs. Future research can investigate this issue. Secondly, regarding the data collection on ESE and entrepreneurs’ ambidextrous behavior, we draw on existing research and use the questionnaire method. This choice is indeed rational, but we recognize that individual cognition is dynamic. Individuals will dynamically adjust their cognition as time and the external environment change. Future research can focus on dynamic changes using panel data to analyze individual cognition. Thirdly, our study investigates conditions in the context of emerging economies represented by China, and it is widely acknowledged that the cultures of different regions will present different individual cognitions. Future research can be extended to other cultural backgrounds to compare the effects of individual cognition on ambidextrous behavior. In addition, given that the need for innovation in high-tech enterprises is more evident and widespread than in other types of companies, the entrepreneurs of high-tech enterprises were selected as research samples. Future research can expand the research object and include other industries and enterprises in the research scope to strengthen the universal applicability of the research. Fourthly, our research focuses on the ambidextrous behavior tendencies of entrepreneurs in new ventures rather than the ambidextrous innovation balance of new ventures. Whether new ventures can use limited resources to achieve an ambidextrous innovation balance and the way to achieve that balance will discuss in our future research.

## Figures and Tables

**Table 1:** Descriptive statistics and Pearson correlations among the variables.

Variables	Mean	s.d.	1	2	3	4	5	6	7	8	9	10	11	12
1. Advanced manufacturing industry	0.463	0.450												
2. High-tech service industry	0.260	0.440	−0.550 **											
3. Other high-tech service industry	0.278	0.449	−0.575 **	−0.367 **										
4. Number of employees	1.098	0.373	−0.076	0.040	0.045									
5. Gender	0.934	0.249	0.140 *	−0.247 **	0.086	0.085								
6. Age	1.612	0.097	0.021	0.087	−0.108	−0.013	−0.138 *							
7. Number of start ups	1.132	0.888	−0.118	0.099	0.035	0.112	0.022	−0.257 **						
8. Time to be an executive	0.849	0.310	−0.068	0.062	0.015	0.049	0.083	−0.082	0.215 **					
9. Economic status	2.918	0.645	0.000	−0.022	0.022	0.413 **	0.117	−0.042	0.112	0.011				
10. Power status	0.419	0.769	−0.050	−0.103	0.157 *	0.055	0.099	0.138 *	0.151 *	−0.143 *	0.109			
11. Self-efficacy	3.102	0.363	0.015	−0.148 *	0.129	0.108	0.196 **	−0.269 **	0.214 **	0.293 **	0.233 **	0.224 **		
12. Entrepreneurs’ ambidextrous behaviour	2.986	0.500	−0.013	−0.048	0.062	0.150 *	0.206 **	−0.270 **	0.322 **	0.038	0.303 **	0.291 **	0.564 **	

* *p* < 0.05, ** *p* < 0.01.

**Table 2:** Results of validity and reliability assessment.

Variables	Cronbach’s α	Kaiser-Meyer-Olkin
Ambidexterity	0.736	0.654
Self-efficacy	0.771	0.666

**Table 3:** Results of regression analyses of entrepreneurs’ ambidextrous behaviour.

Variables	Model 1	Model 2	Model 3	Model 4-1	Model 5-1	Model 6-1	Model 4-2	Model 5-2	Model 6-2
Advanced manufacturing industry	−0.010	0.016	−0.019	−0.019	−0.022	−0.016	−0.002	−0.011	−0.039
High-tech service industry	−0.026	0.060	0.016	0.017	0.011	0.010	0.033	0.027	0.012
Number of employees	0.106 †	0.066	0.048	0.039	0.041	0.043	0.048	0.052	−0.061
Gender	0.166 **	0.109 *	−0.010	−0.010	−0.017	−0.036	−0.016	−0.022	−0.041
Age	−0.177 **	−0.077	−0.114 *	−0.114 *	−0.118 *	−0.108 *	−0.143 **	−0.156 **	−0.156 **
Number of start-ups	0.273 ***	0.214 ***	0.051	0.052	0.054	0.062	0.032	0.029	0.012
Managerial tenure	−0.053	−0.185 ***	−0.009	−0.010	−0.011	−0.020	0.015	0.014	0.036
Self-efficacy		0.532 ***	0.682 ***	0.675 ***	0.667 ***	0.661 ***	0.647 ***	0.667 ***	0.712 ***
Self-efficacy squared			−0.503 ***	−0.496 ***	−0.496 ***	−0.462 ***	−0.493 ***	−0.499 ***	−0.575 ***
Economic status				0.024	0.014	−0.077			
Economic status * Self-efficacy					−0.040	−0.026			
Economic status * Self-efficacy squared						0.143 *			
Power status							0.109 *	0.076	0.162 **
Power status * Self-efficacy								0.078	0.199 **
Power status * Self-efficacy squared									−0.232 **
*R* ^2^	0.185	0.411	0.584	0.584	0.586	0.593	0.593	0.598	0.615
*Adjust R* ^2^	0.159	0.390	0.567	0.565	0.565	0.571	0.575	0.577	0.594
*F*	7.099 ***	19.043 ***	33.849 ***	30.377 ***	27.642 ***	26.034 ***	31.515 ***	29.057 ***	28.541 ***

Note: n = 227 entrepreneurs. † *p* < 0.10, * *p* < 0.05, ** *p* < 0.01, *** *p* < 0.001 (two-tailed).

**Table 4:** Results of sensitivity tests.

	Model						Robustness Check					
Variables	Model 1	Model 2	Model 3	Model 4-1	Model 5-1	Model 6-1	Model 1′	Model 2′	Model 3′	Model 4-1′	Model 5-1′	Model 6-1′
Advanced manufacturing industry	−0.010	0.016	−0.019	−0.019	−0.022	−0.016	−0.008	0.011	−0.020	−0.021	−0.024	−0.011
High-tech service industry	−0.026	0.060	0.016	0.017	0.011	0.010	0.028	0.114	0.059	00.58	00.53	0.060
Number of employees	0.106 †	0.066	0.048	0.039	0.041	0.043	0.121 †	0.092	0.035	0.043	0.045	0.057
Gender	0.166 **	0.109 *	−0.010	−0.010	−0.017	−0.036	0.211 **	0.130 *	0.001	0.001	−0.006	−0.029
Age	−0.177 **	−0.077	−0.114 *	−0.114 *	−0.118 *	−0.108 *	−0.109	−0.051	−0.096 †	−0.096 †	−0.099†	−0.084
Number of start-ups	0.273 ***	0.214 ***	0.051	0.052	0.054	0.062	0.272 ***	0.217 ***	0.074	0.074	0.077	0.095
Managerial tenure	−0.053	−0.185 ***	−0.009	−0.010	−0.011	−0.020	−0.025	−0.203 **	−0.018	−0.016	−0.018	−0.020
Self-efficacy		0.532 ***	0.682 ***	0.675 ***	0.667 ***	0.661 ***		0.539 ***	0.691 ***	0.695 ***	0.688 ***	0.677 ***
Self-efficacy squared			−0.503 ***	−0.496 ***	−0.496 ***	−0.462 ***			−0.486 ***	−0.491 ***	−0.489 ***	−0.435 ***
Economic status				0.024	0.014	−0.077				−0.020	−0.027	−0.139 †
Economic status * Self-efficacy					−0.040	−0.026					−0.031	−0.001
Economic status * Self-efficacy squared						0.143 *						0.177 *
*R* ^2^	0.185	0.411	0.584	0.584	0.586	0.593	0.179	0.406	0.551	0.551	0.552	0.531
*Adjust R* ^2^	0.159	0.390	0.567	0.565	0.565	0.571	0.145	0.377	0.527	0.524	0.522	0.531
*F*	7.099 ***	19.043 ***	33.849 ***	30.377 ***	27.642 ***	26.034 ***	5.249 ***	14.335 ***	22.761 ***	20.388 ***	18.484 ***	17.583 ***

Note: n = 227 entrepreneurs. † *p* < 0.10, * *p* < 0.05, ** *p* < 0.01, *** *p* < 0.001 (two-tailed). Robustness check: Adopting the entrepreneurs’ salary instead of the enterprise registered capital and business turnover in the past year.

## Data Availability

Not applicable.

## Appendix A

No.    

**Questionnaire**
Dear Sir/Madam:
Greeting! We are the graduate students of University of Science and Technology of China. Thank you for sparing time for this questionnaire.
This survey consists of two parts, basic information and investigation and you have 20 min to finish it. This survey is completely anonymous, and your answer is crucial to the final research conclusion. Please fill in truthfully and completely, there is neither right nor wrong, neither good nor bad to your answer. We promise that your answer is only for scientific research purpose, and we will keep it strictly confidential and will not disclose it to any third party. If you are interested in follow-up research progress, we welcome you to contact us.
University of Science and Technology of China
May 2019

**Part 1 Basic Information**
1. Name of the company: 2. Time since established: year(s) 3. Company’s nature: □State-owned □Private-owned □Exclusively Foreign-owned □Joint Venture □Other 4. Main business of the company: 5.Industry: □Electronics and Information Technology □Bioengineering and New Medical Technology □New Materials and Application Technology □Advanced Manufacturing Technology □Aerospace Technology □Modern Agricultural Technology □New Energy and Efficient Energy-saving Technology □New Technology of Environmental Protection □Ocean Engineering Technology □Nuclear Application Technology □Other New Processes and Technologies applied in the transformation of Traditional Industries 6. Company’s Scale (in same industry): □Small □Slightly Small □Middle □Slightly Big □Big 7. Please comment on the market prospect of your main business in the next three years: □Very Bad □Bad □Ordinary □Good □Very Good 8. The average total assets of your company in recent three years is Yuan □Below 5 Milllon □5–40 Million □40–100 Million □100–400 Million □Over 400 Million 9. The average sales volume of your company in recent three years is Yuan □Below 5 Milllon □5–30 Million □30–100 Million □100–300 Million □Over 300 Million 10. The average number of employees in your enterprise in recent three years is □Below 300 □300–1000 □1000–2000 □Over 2500
1. Gender: □Male □Female 2. Age: □25 or Below 25 □26–35 □36–45 □46–55 □56 or over 56 3. Are you the company’s founder: □Yes □No 4. Have you ever started a company: □Yes □No 5. You are engaged in middle and senior positions in the enterprise: Year(s)
1. Registered capital of the company: (in ten thousand Yuan) 2. Are you a representative of the National People’s Congress (national or local): □Yes □No 3. Are you a member of the Chinese People’s Political Consultative Conference (national or local): □Yes □No 4. Your party affiliation: □Communist Party of China □Other □No 5. Your administrative level: □National □Provincial-Ministerial □Bureau-Director or Municipal □Division-Head or County-Head □Section-Head or Township-Section-Head □No 6. Your Education Background: □Junior College or Below □Bachelor □Master □Doctor or above 7. University or College of your highest degree: □Oversea □211, 985 or other key university □Ordinary university or college □Junior College □Other 8. Position you held: □Chairman □General Manager □Deputy General Manager/Branch Manager □Department/Project Leader □Clerk □Other

**Part 2 Investigation**

The following two sets of questions are about your self-efficacy. Please tick the corresponding option according to your own actual situation. The larger the number you choose, the more you agree with this statement. (For first set, 1 = totally disagree, 2 = disagree, 3 = neutral, 4 = agree, 5 = totally agree).

**Question**	**Totally Disagree** **Totally Agree**
1 I feel confident to come up with a new idea through brainstorm.	1 2 3 4 5
2 I feel confident to identify the need for a new product or service.	1 2 3 4 5
3 I feel confident to design a product or service that will satisfy customer needs and wants.	1 2 3 4 5
4 I feel confident to estimate customer demand for a new product or service.	1 2 3 4 5
5 I feel confident to determine a competitive price for a new product or service.	1 2 3 4 5
6 I feel confident to estimate the amount of start-up funds and working capital necessary to start my business.	1 2 3 4 5
7 I feel confident to design an effective marketing/advertising campaign for a new product or service.	1 2 3 4 5
8 I feel confident to get others to identify with and believe in my vision and plans for a new business.	1 2 3 4 5
9 I feel confident to make contact with and exchange information with others effectively.	1 2 3 4 5
10 I feel confident to clearly and concisely explain verbally and write down my business idea in everyday terms.	1 2 3 4 5
11 I feel confident to supervise employees effectively.	1 2 3 4 5
12 I feel confident to recruit and hire employees on my will.	1 2 3 4 5
13 I feel confident to delegate tasks and responsibilities to employees in my business.	1 2 3 4 5
14 I feel confident to deal effectively with day-to-day problems and crises.	1 2 3 4 5
15 I feel confident to inspire, encourage, and motivate my employees.	1 2 3 4 5
16 I feel confident to train my employees.	1 2 3 4 5
17 I feel confident to organize and maintain the financial records of my business.	1 2 3 4 5
18 I feel confident to manage the financial assets of my business.	1 2 3 4 5
19 I feel confident to read and interpret financial statements.	1 2 3 4 5
20 Starting a business is worthless/worthwhile to me. (1 = most worthless, 2 = worthless, 3 = neutral, 4 = worthwhile, 5 = most worthwhile)	1 2 3 4 5
21 Starting a business is disappointing/rewarding to me. (1 = most disappointing, 2 = disappointing, 3 = neutral, 4 = rewarding, 5 = most rewarding)	1 2 3 4 5
22 Starting a business is negative/positive to me. (1 = most negative, 2 = negative, 3 = neutral, 4 = positive, 5 = most positive)	1 2 3 4 5

The following questions are about your ambidextrous behaviors. Please tick the corresponding option according to your own actual situation. The larger the number you choose, the more you agree with this statement. (1 = totally disagree, 2 = disagree, 3 = neutral, 4 = agree, 5 = totally agree).

**Question**	**Totally Disagree** **Totally Agree**
1 I search for new possibilities with respect to products/services processes, or markets.	1 2 3 4 5
2 I evaluate diverse options with respect to products/services processes, or markets.	1 2 3 4 5
3 I focus on strong renewal of products/services or processes.	1 2 3 4 5
4 Exploration activities of associated yields or costs are currently unclear.	1 2 3 4 5
5 Exploration activities require quite some of my adaptability.	1 2 3 4 5
6 Exploration activities of associated yields or costs are currently unclear require me to learn new skills or knowledge.	1 2 3 4 5
7 Exploration activities of associated yields or costs are currently unclear are not (yet) clearly existing company policy.	1 2 3 4 5
8 A lot of experience of exploitation activities has been accumulated by myself.	1 2 3 4 5
9 Exploitation activities I carry out as if it were routine.	1 2 3 4 5
10 Exploitation activities serve existing (internal) customers with existing services/products.	1 2 3 4 5
11 Exploitation activities are clear for me to conduct.	1 2 3 4 5
12 Exploitation activities primarily focused on achieving short-term goals.	1 2 3 4 5
13 I can properly conduct exploitation activities by using my present knowledge.	1 2 3 4 5
14 Exploitation activities fit into existing company policy clearly.	1 2 3 4 5
